# Understanding Trophic Interactions in a Warming World by Bridging Foraging Ecology and Biomechanics with Network Science

**DOI:** 10.1093/icb/icae070

**Published:** 2024-06-13

**Authors:** Jordan P Cuff, David Labonte, Fredric M Windsor

**Affiliations:** School of Natural and Environmental Sciences, Newcastle University, Newcastle-upon-Tyne, NE1 7RU, UK; Department of Bioengineering, Imperial College London, London, SW7 2AZ, UK; School of Biosciences, Cardiff University, Cardiff, CF10 3AX, UK

## Abstract

Climate change will disrupt biological processes at every scale. Ecosystem functions and services vital to ecological resilience are set to shift, with consequences for how we manage land, natural resources, and food systems. Increasing temperatures cause morphological shifts, with concomitant implications for biomechanical performance metrics crucial to trophic interactions. Biomechanical performance, such as maximum bite force or running speed, determines the breadth of resources accessible to consumers, the outcome of interspecific interactions, and thus the structure of ecological networks. Climate change-induced impacts to ecosystem services and resilience are therefore on the horizon, mediated by disruptions of biomechanical performance and, consequently, trophic interactions across whole ecosystems. Here, we argue that there is an urgent need to investigate the complex interactions between climate change, biomechanical traits, and foraging ecology to help predict changes to ecological networks and ecosystem functioning. We discuss how these seemingly disparate disciplines can be connected through network science. Using an ant-plant network as an example, we illustrate how different data types could be integrated to investigate the interaction between warming, bite force, and trophic interactions, and discuss what such an integration will achieve. It is our hope that this integrative framework will help to identify a viable means to elucidate previously intractable impacts of climate change, with effective predictive potential to guide management and mitigation.

## Introduction

Climate change is set to disrupt biological processes at every scale, from the physiology and behavior of individuals (Musolin 2007; Pörtner and Farrell 2008; Travis et al. 2013) to whole ecosystem functioning (Lensing and Wise 2006; Walther 2010; Peters et al. 2013). Changes in environmental conditions such as temperature, barometric pressure, and precipitation have severe consequences for trophic interactions, as they influence their frequencies and identities (Harrington et al. 1999; Winder and Schindler 2004; Blois et al. 2013; Cuff et al. 2023c). Understanding how these interactions shift with weather and climate is consequently important for predicting community-level responses to climatological change (Blois et al. 2013; Singer et al. 2013). Alterations to phenological matches (Thackeray et al. 2016; Renner and Zohner 2018) and species distributions (Poisot et al. 2015) rewire interspecific interactions across entire ecosystems (Winder and Schindler 2004), with cascading effects on ecosystem services and functioning. The implications for trophic interactions will, however, be compounded both directly and indirectly by simultaneous effects on other constraints on foraging ecology, including metabolism (Brown et al. 2004), symbioses (Kikuchi et al. 2016), and biomechanical traits (Domenici and Seebacher 2020). Biomechanics, the physical laws that underpin animal movement and structure, play a key role in determining the feasibility of interactions. Due to their foundation in physics, they are amenable to analysis from first principles, enabling detailed quantitative predictions of relevant performance metrics—a welcome advantage in the otherwise complex and unpredictable context of the effects of climate change on trophic interactions.

Biomechanical traits are crucial determinants of trophic interactions, both directly, for example, through consumer bite forces and resource penetrability (Wang et al. 2017; Püffel et al. 2023b), and indirectly, by determining locomotor capacity or surface attachment forces, which in turn influence predator–prey co-occurrence, and capture and escape efficiencies (Betz and Kölsch 2004; Wilson et al. 2013). Biomechanical properties are, however, modulated by abiotic conditions; for example, higher average temperatures can affect biomechanical performance not only directly (Olberding and Deban 2017, 2021), but also indirectly through changes to animal morphology (Mackenzie et al. 2014; Domenici and Seebacher 2020; Donihue et al. 2020). The body sizes of beetles, for example, may reduce with increasing average temperatures (Tseng et al. 2018) in accordance with the “temperature-size rule” (Klok and Harrison 2013), with likely concomitant consequences for biomechanical performance, such as reduced bite forces (Rühr et al. 2024; Püffel et al. 2023a, 2023b).

**Fig. 1:**
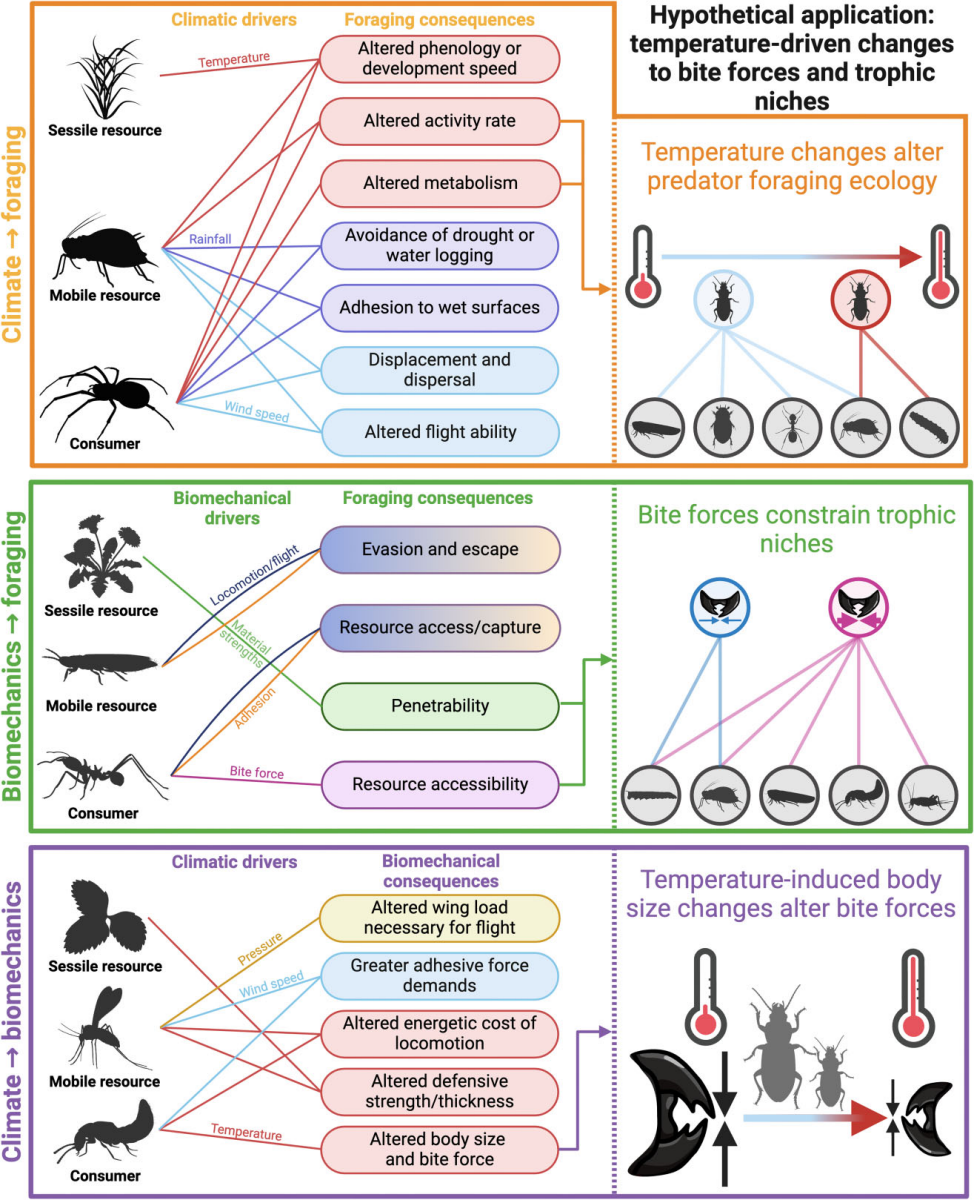
Conceptual diagram pairing drivers with consequences across climate change, biomechanics, and foraging ecology. Outlines of organisms represent different groups and different shaded lines represent different drivers linked to their consequences. The right panel provides an example based on increasing temperature relating to reduced bite forces and therefore reduced access to resources. Climate change impacts foraging dynamics and can consequently cause spatiotemporal changes in trophic interactions. Biomechanical traits determine the feasibility of trophic interactions, with profound impacts on their identities, frequencies, and outcomes. Climate change is set to modify the physiological and morphological parameters that determine the biomechanical performance of organisms, leading to a complex three-way interaction. Network science offers an opportunity to integrate these interactive effects, and to study their combined impacts on trophic interactions. Figure created with Biorender.

The interaction between foraging ecology, biomechanics, and climate change remains poorly resolved, yet the combined impact on trophic interactions has important implications for the ecology of individuals, trophic network structure, and ecosystem functioning. Climate change may consequently disrupt the mechanisms underpinning biological processes as general and significant as conservation biocontrol and species invasions. To understand, predict, and mitigate these effects, the pairwise interactions between climate change, biomechanics, and foraging ecology must first be understood, and then integrated (Figure 1). Here, we describe these interactions and discuss their implications for a range of ecosystem services and ecological phenomena. We argue that integrating foraging ecology, biomechanics, and climate change through network science—the study of interconnected complex systems with graph theory—can clarify the likely impacts of climate change on trophic interactions. To illustrate this idea, we discuss how different data types can be integrated through network science. We also discuss the wider advances that network science might represent in this line of enquiry, and outline the specific requirements of realizing these advances. A glossary of technical terms can be found in the Supplementary Table S1. Appropriate integration of climate change, trophic interactions, and biomechanics has the potential to facilitate not only a deeper understanding of these dynamics but also prediction of future disruptions of ecosystem functioning by climate change.

## Foraging ecology changes with climate

Trophic interactions are driven by a range of processes, including biotic factors, such as resource abundance and choice (Van Der Putten et al. 2010; Vaughan et al. 2018; Cuff et al. 2022b), and abiotic factors, such as altitude, soil, water, and air chemistry, and temperature (Sánchez-Carrillo et al. 2018). Foraging ecology—the study of how animals search for, obtain, and use food resources—accounts for the biotic and abiotic factors that drive these interactions, facilitating investigation of the mechanisms through which climate change will alter them, and the likely consequences of such changes. Climate change will impact foraging ecology via both top-down and bottom-up alterations of ecological interactions. Top-down effects are elicited by changes at higher trophic levels, for example, due to altered predator activity (Lang et al. 2012), metabolism (Csik et al. 2023), or sensory perception (Draper and Weissburg 2019); bottom-up effects are elicited by changes to resources, for example, through alteration of resource growth rate (Went 1953; Ratte 1984), location (Schultz 1998; Ma et al. 2018), or phenology (Forrest 2016; Renner and Zohner 2018). Irrespective of their directionality, these alterations may alternatively be broadly categorized as ecological (e.g., phenology, co-occurrence) or physiological (e.g., metabolism, sensory perception; Laws 2017).

The ecological impacts of climate change on foraging ecology can be both direct and indirect; indirect effects are typically either spatial (e.g., resource distribution) or temporal (e.g., phenology; Figure 1; Van Der Putten et al. 2010; Thackeray et al. 2016; Renner and Zohner 2018; Cuff et al. 2023a). Resource community dynamics and consumer-resource co-occurrence are easily observable manifestations of environmental change (Van Der Putten et al. 2010; Renner and Zohner 2018). For plant–animal interactions, climate change can alter co-occurrences by altering the emergence time of animals or the development time of plants, with mismatches between resource availability and consumer activity resulting in few or poor-quality resources (Feeny 1970; Hunter 1990; Singer and Parmesan 2010; Kharouba et al. 2015; Ross et al. 2017). For predator–prey interactions, structural changes in prey communities can result from migration of prey in response to environmental change (Van Der Putten et al. 2010; Yang et al. 2011), changes in prey behavior (Knowlton and Graham 2010; Harborne 2013), or survival of prey in environmental extremes (Aleuy and Kutz 2020; Neilson et al. 2020), all of which can result in large fluctuations in predator–prey co-occurrence rates. Environmental variation can also change co-occurrence patterns more directly; for example, greater wind speeds may dislodge animals (Donihue et al. 2020), complicate foraging by aerial predators (Lane et al. 2019), or make the transport of resources more costly (Alma et al. 2016a, 2016b). Although consumer-resource co-occurrence is a necessary condition for trophic interactions, it does not determine them outright (Blanchet et al. 2020). Prey preferences of predators can change irrespective of prey availability (Cuff et al. 2022b), perhaps dictated by metabolic, and other physiological changes (Jackson et al. 2004).

Climate change also brings about physiological changes, which usually affect trophic interactions indirectly. Higher temperatures increase metabolic rates, leading to greater energy demands and a concomitant increase in feeding rates (Robinson et al. 1983; Gillooly et al. 2001; Boscolo-Galazzo et al. 2018; Csik et al. 2023), which may manifest in greater consumer generality (Cuff et al. 2023c) or changes in resource choice (Eitzinger et al. 2021). This could rewire trophic networks and increase competition for high-quality resources (Lang et al. 2012). Heightened metabolic demands with higher temperatures may also increase animal activity (Gibert et al. 2016; Terlau et al. 2023), but it can also decrease in small animals (Lang et al. 2012); regardless, variation in activity will alter co-occurrence rates and, consequently, the frequencies and identities of interactions. The production, emission, and detection of sensory stimuli, crucial determinants of trophic interactions, is also impacted by temperature (Tingey et al. 1980; Peñuelas and Staudt 2010; Sentis et al. 2015), humidity (Menzel et al. 2018; Sprenger et al. 2018; Baumgart et al. 2022), and wind (Goldansaz and McNeil 2006; Hall et al. 2012; Wijers et al. 2022). Although its impact is both broad and significant, animal ecology is not the sole determinant of trophic interactions. Instead, it will interact with biomechanical constraints to determine trophic interaction identities, frequencies, and outcomes.

## Biomechanical traits constrain foraging ecology

Physical processes play a key role in animal foraging, and biomechanical traits have thus long been an important element of foraging theory (Stephens and Krebs 1986; Domenici 2001; Combes et al. 2012; Moore and Biewener 2015). The role of biomechanical traits in foraging is, with few exceptions, eventually linked back to the performance of muscle, the primary agent of motion. As a broad characterization, biomechanical constraints on muscle may be placed in one of two categories: dynamic constraints, which determine energy output during continuous movements or explosive predatory strikes; and effectively quasi-static constraints, which determine the magnitude of the maximum forces animals can apply (Alexander 2006).

Dynamic biomechanical constraints typically play a key role during resource acquisition, as is perhaps most evident in predator–prey interactions that involve pursuit. Successful pursuit hinges on a complex trade-off between speed, maneuvrability, and predictability (Domenici 2001; Combes et al. 2012; Wilson et al. 2013, 2018; Clemente and Wilson 2015; Moore and Biewener 2015; Martin et al. 2022); for example, cheetahs run at sub-optimal speeds in the pursuit of agile prey in order to retain maneuvrability (Wilson et al. 2013), and fruit flies maximize their escape chances when increasing the frequency of erratic saccade maneuvers (Combes et al. 2012). Although biomechanical constraints are not the sole determinant of the outcome of trophic interactions (Martin et al. 2022), they can drive musculoskeletal adaptations over both evolutionary and developmental time scales. For example, predator–prey interactions can place high athletic demands on predators, which consequently exceed their prey in muscle power, acceleration, and deceleration capacity, such as in big cats on the savannah (Wilson et al. 2018). Similarly, grasshoppers reared in the presence of predatory threats adjust their jumping “technique” such that they can jump quicker and further compared to conspecifics raised in safe environments (Hawlena et al. 2011).

Quasi-static biomechanical constraints come into play primarily during resource consumption: in both invertebrates and vertebrates, bite forces play a key role in foraging because they determine the type and size of food items animals can mechanically process (e.g., Wheater and Evans 1989; Behrens Yamada and G. Boulding 1998; Herrel et al. 2001; Schenk and Wainwright 2001; Verwaijen et al. 2002; Christiansen and Wroe 2007; Santana et al. 2010; Tan et al. 2021; Püffel et al. 2023c). Bite forces thus also influence interspecific competition, for example, in predator–predator interactions (Ginot et al. 2018), or in controlling resource accessibility. Some weevils escape predation by tree lizards because they are significantly harder to break open than comparable prey in their habitat (Wang et al. 2017), and the strong bite forces of predators may have been a contributing factor to the evolution of exceptional toughness in mollusc “armour” such as nacre (Jackson et al. 1988). Because sufficiently high bite forces are a necessary condition for successful feeding in many animals, dietary needs have been explicitly argued to have driven the evolution of bite performance in reptiles (e.g., Metzger and Herrel 2005; Dollion et al. 2017), benthic predators such as crabs (Taylor 2000), and phytophagous insects such as leaf-cutter ants (Püffel et al. 2023b). Indeed, bite forces in some amniote groups have evolved through bursts of exceptional rates of adaptive change (Sakamoto et al. 2019).

Force capacity is also important for a range of specialized behaviors that involve surface attachment. For example, palmetto tortoise beetles produce attachment forces so large that predatory ants fail to prey on them (Eisner and Aneshansley 2000); pitcher plants trap insects with pitfall traps that make them slip (Bohn and Federle 2004; Labonte et al. 2021); and specialized adhesive organs play an important role in prey capture and predator defense in invertebrates, vertebrates, and plants (e.g., Herrel et al. 2000; Betz and Kölsch 2004; Poppinga et al. 2012; Krimmel and Pearse 2013; Wolff et al. 2014, 2017; Kleinteich and Gorb 2015; Von Byern et al. 2017). Here, force capacity may be determined primarily by the stress capacity and size of adhesive patches (Labonte and Federle 2015), instead of animal muscle force (but see Labonte et al. 2019). Although biomechanical constraints on foraging ecology are in principle measurable and often even predictable via first principles, the underlying parameters are not impervious to external influence. How will climate change confound and perturb biomechanical constraints?

## Climate change modifies biomechanical performance

Given the broad range of meteorological impacts associated with climate change, the biomechanical performance of animals will be challenged on many fronts (Domenici and Seebacher 2020). Variations in environmental conditions due to climate change can both directly and indirectly alter biomechanical performance through a range of mechanisms, acting across different timescales.

Direct impacts encompass immediate fluctuations in external biomechanical demands and in intrinsic biomechanical performance capacity; for example, variations in wind or water currents can increase the external demand placed on adhesive organs (Forrester et al. 2016; Cherry and Barton 2017; Donihue et al. 2020) or hinder locomotion (Kramer and McLaughlin 2001; Cherry and Barton 2017; Ventura et al. 2022). Intrinsic locomotor performance capacity is determined by muscle (Biewener 2016), and the mechanical performance of muscle, including muscle shortening speed and power output, approximately doubles for every 10°C of temperature increase (i.e., the Q_10_ is ∼2), until performance eventually plateaus at high temperatures (Bennett 1985, 1990; James 2013; Olberding and Deban 2017; James and Tallis 2019). Consequently, maximum running speed has a Q_10_ of around two in both ecto- and endotherms for temperatures in the broad range of 10–50°C (Bergmann and Irschick 2006; Hurlbert et al. 2008; Rojas et al. 2012), equivalent to a 10% increase in maximum speed per degree Celsius warming. The downstream effects of such fluctuations on predator–prey interactions may thus be substantial (e.g., James 2013; Domenici et al. 2019), but remain difficult to predict because the variation of maximum speed with body size is non-monotonous (Garland 1983; Hirt et al. 2017; Labonte et al. 2024). In addition, locomotion at higher temperatures may become more costly, hinting at the possibility of complex trade-offs (Halsey 2016; Seebacher et al. 2016). Available evidence suggests that the phenotypic plasticity to cope with temperature-induced fluctuations in locomotor performance capacity is limited; it appears that the thermal sensitivity of the chemical processes that underpin muscle contractions cannot be avoided (Bennett 1985, 1990; see James 2013; James and Tallis 2019). However, locomotor strategies that are based on temperature-insensitive mechanical processes do exist: many small animals temporarily “store” muscle work in latched “springs” in the form of elastic strain energy. This energy is then released rapidly to drive explosive jumps. Because muscle force capacity tends to only show a weak temperature dependence (Bennett 1985, 1990; Olberding and Deban 2017), such spring-actuated jumping can be “thermally robust” (Olberding and Deban 2021).

Indirect impacts of climate change on biomechanical demands and performance capacity may emerge through adaptive processes, and thus occur on substantially longer time scales. The most significant adaptation to a warming climate may be variations in animal body size (Atkinson 1994; Sheridan and Bickford 2011; Tseng et al. 2018). Body size is a major predictor of a range of biomechanical performance metrics, including maximum running speed and force capacity (Schmidt-Nielsen 1984; Alexander 1985; Biewener 2005), but also for structural traits such as rigidity and the external loads that can be sustained without material failure (Alexander 1981). The relationship between body size and biomechanical capacity is often regular, so that it can be described to reasonable accuracy through statistical analysis, and it is also usually the result of first-principle physical constraints, so that it can be linked with measurable phenotypic traits in predictive models (Schmidt-Nielsen 1984). The relation between trait *T* and body mass *m* is typically expressed via power laws: *T ∼ m^x^*, where *x* is a characteristic scaling coefficient. The immediate advantage of such regularity is that it provides the possibility to link temperature-induced changes in body size with variations in biomechanical performance. If body sizes decrease by a factor α with every degree Celsius, then the biomechanical performance decreases by a factor *α^x^*. Thus, for a decrease in body size of about 10% (*α* = 0.9), *T* would consequently decrease by 3–10%, for traits that scale with length (*x* = 1/3), area (*x* = 2/3), or volume (*x* = 1). The utility of such simple calculations is illustrated with a concrete example further below.

Environmental change can also impact structural and morphological traits independent of muscle physiology or variations in body size, with downstream effects on locomotion, predation efficiency, or defense. For example, an increase in temperature can result in nutritional deficits or allocation trade-offs in which energy and nutrients are diverted toward the mitigation of the physiological impacts of warming, at the expense of defensive traits (e.g., shell or chitin thickness; Mackenzie et al. 2014; Woodman et al. 2021). More generally, the mechanical properties of biological materials, often considered extended phenotypes, can also be sensitive to fluctuations in temperature and humidity (e.g., in spider silk; Blamires et al. 2017; Blamires and Sellers 2019). Adaptive and evolutionary processes can drive biomechanical changes, and organisms may be able to compensate for and acclimatize to environmental change to some extent (Le Roy et al. 2017), potentially buffering biomechanical and ecological impacts. However, the plasticity of species and specific traits to change can vary greatly (Padilla et al. 2019) and will ultimately impact the ability of animals to forage, resulting in a complex three-way interaction between foraging ecology, biomechanics, and climate change.

## Integrating foraging ecology, biomechanics, and climate through network science

We have discussed the impact of climate change on both biomechanical performance and foraging ecology, and how biomechanics interacts with foraging ecology to determine trophic interactions. This complex three-way interaction (Figure 1) likely affects several key biological processes: biocontrol of crop pests by predators, and species invasions offer just two striking examples in which ecosystem services and functioning may be substantially perturbed by the emergent effects of climate change on biomechanical traits and foraging (Table 1). Studying this three-way interaction remains challenging, partly due to the innate transdisciplinarity required to do so successfully. Foraging ecology provides observational data on trophic interactions from laboratory feeding trials and/or the field, which can serve as a baseline for comparison against biomechanical performance; biomechanical research provides quantitative mechanistic context to interaction data, usually derived from laboratory assays or from predictive physical models derived from first principles. Integrating these practices in climatological contexts requires records and predictions of climatological conditions, and empirical or theoretical models that encode their impact on foraging or biomechanical traits. This process will naturally require adherence to disparate or even conflicting best practices; for example, the requirement for fresh contaminant-free samples for molecular dietary analyses (increasingly commonplace for detecting trophic interactions in ecology; McInnes et al. 2017; Alberdi et al. 2019; Cuff et al. 2023a), may conflict with the need to measure bite forces from the same individuals. The collection of some data in the field (e.g., ecological observations) and others within the lab (e.g., biomechanical assays) is often necessary, but may result in temporal or contextual mismatches due to unavoidable differences in experimental conditions (Campbell et al. 2009). Developing biomechanical methods that can be brought to the field (Bauer et al. 2020) is one of many vital ways forward that may help resolve such discrepancies.

**Table 1:** Various ecological systems, processes and services will be disrupted by climate change due to associated and interactive ecological and biomechanical changes. Biological control and species invasions/reintroductions offer two examples of systems that transdisciplinary research spanning these fields could address. Various hypotheses remain to be tested for this interaction between foraging, climate and biomechanics.

System	Climatic driver	Consequence	Impact	Relevant references
Biological control	Temperature changes	Biomechanical: bite force changes with altered body size	Ecological: trophic niche contraction as resources become inaccessible	Tseng et al. (2018); Rühr et al. (2024); Püffel et al. (2023c)
		Ecological: phenological changes causing pest-predator phenological mismatches	Biomechanical: trait mismatch between consumers and available resources	Singer and Parmesan (2010); Thackeray et al. (2016); Renner and Zohner (2018)
	Wind/Pressure changes	Biomechanical: altered flight ability of many crop pests and predators	Ecological: altered co-occurrence of consumers and resources	Walters and Dixon (1984)
		Biomechanical: increased adhesive forces required or dislodgement		Forrester et al. (2016); Cherry and Barton (2017); Donihue et al. (2020)
Species invasions and reintroductions	Temperature changes	Ecological: invasive range changes	Biomechanical: altered access to resources and evolutionary mismatch between consumer and resources	Hellmann et al. (2008); Smith et al. (2012); Zhang et al. (2020); Rühr et al. (2024)
		Biomechanical: bite force reduction with smaller body size	Ecological: a species’ invasive potential may be reduced if their access to resources is hindered	Tseng et al. (2018); Püffel et al. (2023c)
	Wind/Pressure changes	Biomechanical: altered flight/dispersal ability	Ecological: altered dispersal potential	Saura-Mas and Lloret (2005); Lander et al. (2014)

One analytical, predictive, and therefore attractive framework that can integrate ecological, biomechanical, and climatological data is network science—the analysis of interconnected complex systems. The application of network science to ecological networks principally requires two data types: links and nodes (Fath et al. 2007; Lau et al. 2017). Typically, links are the presence or frequency of interactions between organisms; nodes usually represent species identities, but can also represent individuals (Guimarães 2020) or even trait data such as morphological features or environmental context (Poisot et al. 2015; Cuff et al. 2023c; Cuff et al. 2022a). Through the integration of biomechanical data as traits attributed to nodes (i.e., species or groups of individuals with similar biomechanical performance/traits), foraging data as links between those nodes (i.e., trophic interactions) and climate data as spatiotemporal replication (i.e., discrete networks based on climatic differences), network ecology may enable exploration of how climate drives trophic interactions via biomechanical changes. Although the same could be achieved through methods such as fourth-corner analysis, which relates species traits to the relationship between community data and environmental variables, this neglects indirect interactions and ignores the structure of interaction networks, both crucial for understanding interaction rewiring and network robustness. With appropriate data, such an approach could help to identify how climate change impacts the structure of networks by revealing differences in the interactions between animals based on their changing biomechanical performance.

The same idea can be applied to key ecological processes like ecosystem services (Dee et al. 2017; Bodin et al. 2019) and nutritional cascades (Cuff et al. 2022c) for deeper ecological and applied insight. There is a hierarchy of structural properties that can be analyzed and compared at the node (e.g., species or individuals, studied in biomechanical assays), group (e.g., trophic levels, studied in foraging ecology), or network (e.g., habitat or ecosystems, subject to climate change impacts) levels (Lau et al. 2017). By analyzing the spatiotemporal fluctuation of networks, it is possible to determine intuitive parameters with immediate management implications, such as network robustness (i.e., the rate of secondary extinctions when some nodes/species are removed; Kaiser-Bunbury et al. 2010; Pocock et al. 2012). The results can be used, for example, to determine any indirect impacts on ants as a result of climate change-mediated removal of the epiphytes they depend on, and vice versa (Morales‐Linares et al. 2021); through integration of biomechanical data, the changing accessibility of resources could be considered as primary extinctions in the network.

Biomechanical properties can be integrated into networks as traits (Eklöf et al. 2013; Junker et al. 2013; Poisot et al. 2015), as previously demonstrated for body size (Woodward et al. 2005). By representing individual organisms through their biomechanical traits, structural differences in interspecific interactions can be illuminated, and the mechanisms by which biomechanical traits structure interactions elucidated. This process can, for example, lead to the identification of motifs (i.e., sets of nodes with similar interactions) that are associated with particular biomechanical properties. For example, predators with weaker bite forces may interact consistently with more penetrable prey. Identification of such correlations would allow for a more directed monitoring of the response to environmental change. Motifs also facilitate investigation of indirect interactions (e.g., the role of plants in supporting predators/parasitoids; Tavella et al. 2022), which remain biomechanically poorly understood.

By representing different time points, spaces, or data types as distinct network layers, further complexity can be modeled. Given the complex interdependencies of different biomechanical properties and ecological processes, multilayer networks present an opportunity to link discrete networks across space and time in response to changing environmental conditions (Pilosof et al. 2017; Hutchinson et al. 2019). For example, networks could each represent climatically distinct time points and the interlayer links (i.e., the links between these networks) could represent changes in the biomechanical performance of the animals within the networks; this could more directly elucidate the impact of these biomechanical changes on network structures across time.

## The potential of network analysis to investigate the impact of temperature increases on trophic interactions as a consequence of bite force changes

**Fig. 2:**
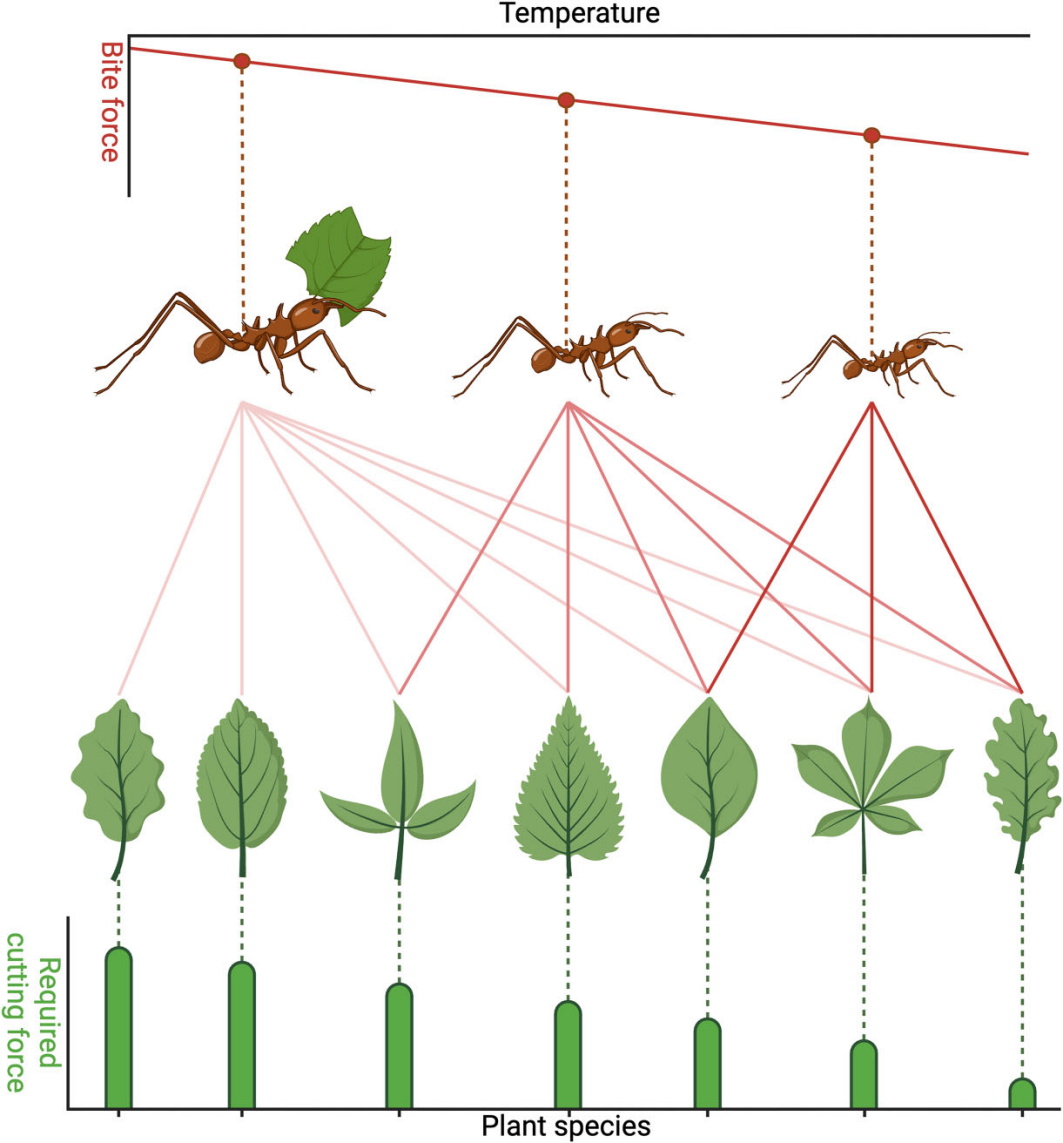
We hypothesize that, as temperatures increase, ants may undergo body size reductions in line with the temperature-size rule. Because body size correlates with bite force capacity, and because bite force capacity determines the toughest plant tissue the ants can cut, this size reduction would likely decrease the total number of plant species the ants can forage on (i.e., contract their trophic niche). Figure created with Biorender.

The integration of biomechanics, foraging ecology, and climate change through network science has the potential to elucidate the mechanisms by which trophic networks will respond to climate change, ultimately enabling us to understand and predict these impacts, which we illustrate with a simplified illustrative example (Supplementary Information 1). Network science generally requires data on interaction identities, interaction rates between consumers and their resources (i.e., weighted links), and ideally resource abundances, all in discrete spatiotemporal units. To the best of our knowledge, such comprehensive data are currently seldomly available in biomechanical contexts and will require targeted data collection efforts. To illustrate how such data could be integrated to address fundamental questions, we present a concrete example where sufficient data are available or can be generated to illustrate the main idea within a specific context: the change in resource accessibility for a generalist insect herbivore due to climate change-induced reductions in body size (Figure 2). This example is not meant to be read as a concrete prediction. Instead, it is intended to provide an indication of both the process and the kind of information that can be extracted by combining climate data, foraging ecology, and biomechanical traits through network science.

We have compiled leaf-cutter ant body size and bite force data for three colonies of the same species from Püffel et al. (2023c), required cutting forces for a range of plants globally available to the ants from Onoda et al. (2011), and insect temperature-body size relationships from Tseng et al. (2018). To provide a practical example of how network science can enable direct predictions of the effect of climate change on trophic interactions with a strong biomechanical component, we constructed networks for different temperatures using these data (Supplementary Information 1). These networks represent the interactions possible for the ants within the biomechanical constraints imposed by the bite forces they can generate. However, because not all plant species for which data exist will co-occur, and because the ants may exhibit selectivity across the plants accessible to them, this is not a direct reflection of the interactions within a natural system. Nevertheless, analysis of the degree (i.e., number of plants each ant colony could interact with) and generality (i.e., the potential niche breadth of ants generally) of the leaf-cutter ants in response to temperature will give some indication of how interactions may change in smaller discrete communities of ants and their resources. With data on those discrete communities and wider interactions of other consumers and their antagonists, network science would be poised to illuminate competitive dynamics, indirect interactions, and ecosystem-wide cascades.

This example is limited to a simple network analysis by necessity—the data are currently scarce. Richer datasets, purposefully collected to enable network analyses, may reveal real-world effects, but the collection of such data requires careful consideration, which we hope our article can guide. The example also assumes that the temperature-size rule is robust and the major driver of network rewiring, neither of which are likely to hold in natural systems. For example, alterations in the structure of plant–ant networks may concentrate herbivory on fewer plant species, increasing competition and reducing availability of those resources. Temperature increases also have various implications for the trophic interactions of ants, including altered search behavior (Frizzi 2018) and foraging site selection (Traniello et al. 1984; Spicer et al. 2017), which will interact with the temperature-size rule and likely alter predictions. The selective pressure linked to the temperature-size rule is also noteworthy; the reduced access to resources through smaller body sizes may well propagate a selective pressure for larger body sizes that will potentially mitigate the impacts of the temperature-size rule in natural systems. These limitations warrant skepticism, and more accurate models will have to await the availability of purposefully collected data.

The species turnover within natural systems, both naturally over time and due to climate change, will also greatly impact outcomes. Climate change-induced changes to other biological and ecological properties will also compound predictions; for example, reduced defoliation of a plant species may increase its fitness by relief of damage or the cost of induced defenses (Karban and Myers 1989), and may consequently alter that species’ abundance, biomass, and range/distribution. Species turnover will likely be driven by the population dynamics of each species within a community; by evaluating changing interactions across entire ecosystems, network ecology is well-placed to assess how the turnover and dynamics of species will affect wider interactions through network robustness and rewiring analyses. Other drivers and constraints of trophic interactions, such as the chemical composition of leaves and the nutritional quality and complementarity of available resources, will likely change markedly too, which are, of course, not represented within our analysis. The wider effects of climate change on broader ecological phenomena may only be captured by real-world data. With appropriate information on other interspecific interactions of both ants and the plants they forage on (e.g., symbionts, predators, and parasites), these impacts can be mapped across the entire ecosystem (Windsor et al. 2023), a distinct benefit of taking a network approach to such ecological questions.

Knowledge of interaction strengths would enable a range of further quantitative analyses. First, we may predict how trophic interactions and entire networks rewire in response to critical events, be it invasion by a new resource or consumer species, or changes in the biomechanical properties of either group due to environmental change. Using trait matching approaches, which investigate how traits determine the likelihood of interaction (Pichler et al. 2020), the new interactions that arise following disturbances could be predicted. Second, the network structure itself can be used to estimate the system’s resistance and resilience through methods such as network robustness analyses, which assess the rate of secondary extinctions in a network following the removal of nodes (e.g., Kaiser-Bunbury et al. 2010; Pocock et al. 2012). Such methods explicitly consider the ability of networks to respond to events at the whole system level and can help identify specific organisms that act as ecosystem stabilizers (Harvey et al. 2017).

Several key hypotheses emerge through consideration of our illustrative example. The core hypotheses of our example are among these: (i) temperature increases will decrease the generality of ants; (ii) temperature increases will decrease the degree of individual ant colonies (i.e., the diversity of plants they interact with); and (iii) the ant degree change will differ between colonies depending on their initial bite forces. Our results present a hypothetical prediction, based on the assumption that biomechanical traits are indeed the primary driver of climate change-induced changes to trophic interactions. This idealized expectation is ideal for comparison against real-world data. As discussed above, by virtue of being illustrative and idealized, the analysis has omitted several other variables that may exert an influence, including how the penetrability of resources (e.g., the cutting force required to break leaves) may change. Testing the hypotheses outlined for our example, however, requires repeated measurements over long time series across large spatial scales and, ideally, with other driving factors accounted for (e.g., leaf chemistry and penetrability, ant activity rates, and metabolism). The case for pursuing this is nevertheless strong given the profound implications such changes may have on ecosystem-wide dynamics.

## Summary

Foraging ecology, biomechanics, and climate change likely influence the frequency and identity of trophic interactions interactively and dynamically, but direct evidence for such effects remains scarce and disjointed. The potential implications for ecosystem services like biocontrol and global challenges like species invasions are profound, and will likely intensify in the coming decades, rendering directed research efforts that explore this disciplinary interface valuable. Understanding and predicting these impacts will be crucial for maintaining healthy food systems and ecosystems, and urgently require transdisciplinary research using convergent approaches. Network science has the potential to illuminate the mechanisms by which biomechanical traits and foraging ecology structure trophic networks in response to climate change, and may provide much-needed predictions of responses to projected change, but obstacles still exist.

## Author contributions

J.P.C.: Conceptualization; Writing – original draft; Writing – review and editing; Visualization; Formal analysis. D.L.: Conceptualization; Writing – original draft; Writing – review and editing; Formal analysis. F.M.W.: Conceptualization; Writing – original draft; Writing – review and editing; Visualization; Formal analysis

## Supplementary Material

icae070_Supplemental_File

## Data Availability

All data and code used in this manuscript are openly available via Zenodo: 10.5281/zenodo.10284904 (Cuff et al. 2023b).
