# Supplementary material for: Understanding Trophic Interactions in a Warming World by Bridging Foraging Ecology and Biomechanics with Network Science
Source: Integr Comp Biol. 2024 Jun 13;64(2):306–21. doi: 10.1093/icb/icae070 (PMC11406160; doi:10.1093/icb/icae070)
Supplement: icae070_Supplemental_File [file icae070_supplemental_file.docx]

**Understanding trophic interactions in a warming world by bridging foraging ecology and biomechanics with network science - Supplementary Information**

**Supplementary Information 1: Data processing, visualisation and analysis for an illustrative example**

***Background***

Leaf-cutter ants (*Atta* spp. and *Acromyrmex* spp.) are the principal insect pest and a major ecosystem engineer throughout the Neotropics (Leal et al., 2014; Wirth et al., 2003). They harvest plant matter in the surroundings of their colonies to grow a fungus as crop, and in doing so they cut plant matter on an almost industrial scale: about 15 % of the foliar biomass in the Neotropics, or about every sixth leaf, is consumed by leaf-cutter ant colonies (Costa et al., 2008; Fowler et al., 1989; Herz et al., 2007; Wirth et al., 2003), and more than half of all woody species are attacked by them (Cherrett, 1968; Rockwood, 1976). Leaf-cutter ants are perhaps the most voracious and polyphagous herbivorous insects (Lugo et al., 1973; Wirth et al., 2003), and their foraging activity is affected by a variety of environmental conditions, including wind (Alma et al., 2016b), precipitation (Steadman et al., 2020) and barometric pressure (Sujimoto et al., 2020), all of which will be subject to variation due to climate change.

Although leaf-cutter foraging is clearly a complex, multi-factorial behaviour, it has at its core a biomechanical interaction between ant consumer and plant food resource: the force the ants can apply must exceed the force required to drag the mandible through the tissue (Püffel, Roces, et al., 2023; Püffel, Walthaus, et al., 2023). The magnitude of the available bite force is determined by worker size, and the magnitude of the minimum required cutting force is determined by structural and mechanical properties of the plant leaf; consumer and resource properties interact. This mechanical competition has resulted in extraordinary adaptations in both the anatomy and physiology of the leaf-cutter ant bite apparatus: their disproportionately large heads are filled to the rim with optimally packed mandible closer muscles (Püffel et al., 2021). Both their muscle stress and size-specific bite forces are among the highest measured for any animal (Püffel, Johnston, et al., 2023; Püffel, Roces, et al., 2023), and their mandibles are close to “ideally sharp” (Püffel, Walthaus, et al., 2023). As a result, the vast majority of worker sizes can cut the majority of tropical leafs; without these adaptations, and a bite performance commensurate with their body size, only the largest workers would be able to perform this crucial mechanical task (Püffel, Roces, et al., 2023). How will a warming climate affect resource accessibility for the leaf-cutters?

Temperature increases have various implications for the trophic interactions of ants, including altered search behaviour (Frizzi, 2018), and foraging site selection (Spicer et al., 2017; Traniello et al., 1984). An increase in average temperatures can also drive body size decreases in insects (Tseng et al., 2018), including ants (Molet et al., 2017)[,](https://www.zotero.org/google-docs/?broken=QmLD4C) concomitantly reducing their available bite force (Püffel, Roces, et al., 2023; Rühr et al., 2022). Since leaf-cutter mandibles are so sharp that they already cut with a force close to the minimum dictated by cutting mechanics, the force required to cut leaves will likely be unaffected (Püffel, Walthaus, et al., 2023), and any change in body size will therefore only significantly impact bite forces. Because the relationship between bite forces and body size in the leaf-cutter is well understood mechanistically (Püffel, Roces, et al., 2023), it is possible to predict how these changes will impact trophic networks. A very rough estimate of the change in network structure serves to illustrate how network science can integrate biomechanics and foraging ecology to study the effect of climate change on trophic interactions.

To demonstrate the potential of network science to integrate biomechanical and foraging data within the context of climate change, we constructed and analysed hypothetical plant-ant networks across six hypothetical temperatures. This is framed around testing the following hypotheses: (i) temperature increases will decrease the generality of ants; (ii) temperature increases will decrease the degree of individual ant colonies (i.e., the diversity of plants they interact with); and (iii) the ant degree change will differ between colonies depending on their initial bite force.

***Datasets and methods***

All analysis was performed in R version 4.3.1 (R Core Team, 2023), and data processed reproducibly via the ‘tidyverse’ package (Wickham et al., 2019). We compiled two datasets and some additional contextual information. Leaf-cutter ant biomass (a proxy for body size) and bite force data were taken from Püffel et al. (2023) for 248 individual ants across three colonies. Required cutting forces for 1197 individual plants representing 868 taxa available to leaf-cutter ants were taken from Onoda et al. (2011). Insect temperature-body size relationships were taken from Tseng et al. (2018); specifically, a body size decrease of 1.56 % per degree Celsius increase for museum specimens, to represent gradual long-term change. Based on these data, edgelists (i.e., pairwise lists of consumers and resources) were generated for ants and plants in which binary interaction weights were applied; where bite forces exceeded the force required to cut leaves, a weighting of 1 was given, and 0 otherwise. This edgelist was then replicated for incremental increases of 1 °C up to a 5 °C increase by adjusting bite forces based on incremental body size decreases of 1.56 %. To estimate the change of bite force with body mass, we used direct bite force measurements from Püffel et al. (2023), which suggest that maximum bite force in *Atta vollenweideri* varies with body mass *m* as *T ~ m^0.9*. Thus, if body size decreases by a factor of 0.9844 (i.e., 1.56 % decrease) with every degree Celsius temperature increase, then the maximum bite force decreases by a factor of 0.9844*^0.9^*. Consequently, adjusted bite forces were calculated, and new binary edgelist weightings generated based on whether the adjusted bite force was greater than the required cutting force.

Bipartite networks were constructed with consumer nodes and resource nodes representing the three ant colonies and the 868 plant taxa, respectively. All six networks were visualised using ‘ggnetwork’ (Briatte, 2021) via ‘igraph’ (Csardi & Nepusz, 2006) in a single network diagram to highlight persistence of links across temperatures using scaled red colours. Network metrics, specifically consumer degree (the number of plants ants were deemed able to interact with) and generality (the total range of plants accessible across all ants), were generated via the ‘bipartite’ package (Dormann et al., 2008) and visually compared via ‘ggplot2’ (Wickham, 2016).

***Data and outcomes***

Across worker ants in the three ant colonies represented in the ant data from Püffel et al. (2023), the ants could in principle cut the leaves of about 90.05 % of the 868 tropical plant species for which data were available (Onoda et al., 2011). Assuming a decrease in (dry) body mass of 1.56 % per degree Celsius warming, as observed in museum specimen beetles (Tseng et al., 2018), a 2 °C increase would decrease the body mass of a 5 mg forager to 4.85 mg (5(1-0.0156)^2^), and the maximum bite force would decrease accordingly (x((1-0.0156)^0.9^)^2^). Across the three ant colonies, the fraction of cuttable leaf laminas is reduced to about 89.71 % (a mean loss of 3 potential resource species); for a 5 °C increase, this further reduces to 89.13 % (a loss of 8 resource species from baseline). In other words, every degree of warming reduces the percentage of accessible species by about 0.18 %. A warming climate will likely affect both the generality (i.e., the niche breadth) and the degree (i.e., the number of accessible plant species) of leaf-cutter ant colonies (Figure S1).


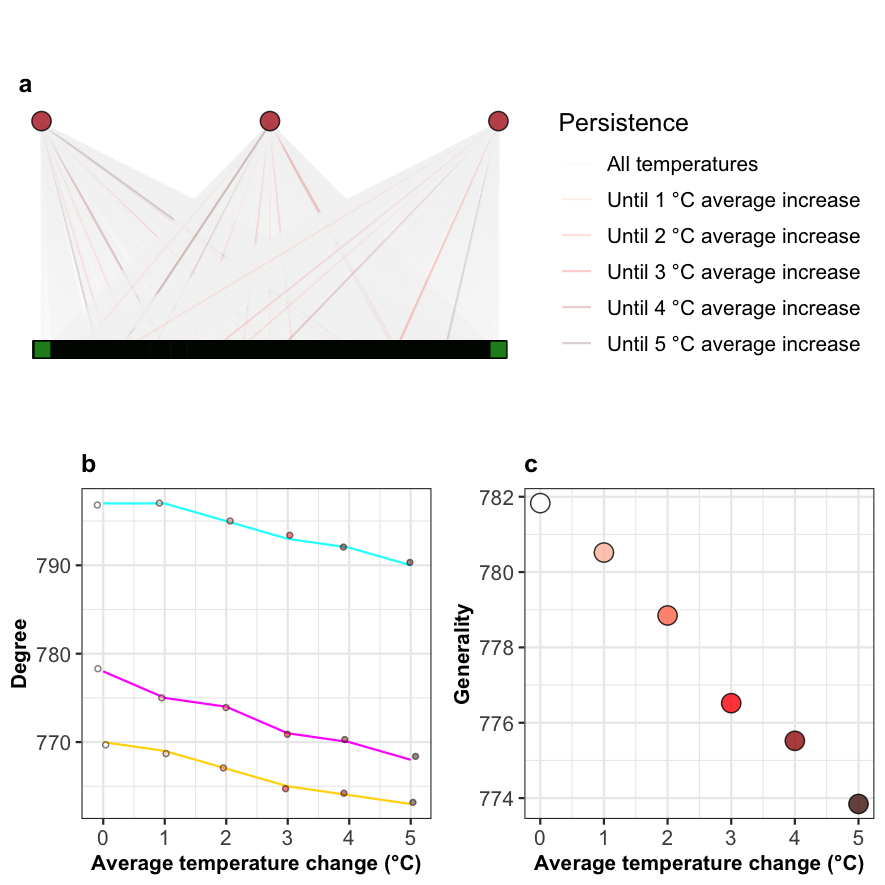


**Figure S1.** Variation in the network of plant-ant interactions in response to average temperature changes. a: The plant-ant network; upper nodes represent three distinct leaf-cutter ant colonies for which bite forces varied, and lower nodes represent plant species. Grey links denote interactions feasible at all considered temperatures, based on projected body size and bite force (see text for details). Scaled red links denote the loss of interactions with incremental average temperature increases. b: The node degree (i.e., hypothetical number of plant species for which leaves are accessible) of leaf-cutter ants from three colonies (line colours) from which bite force measurements were taken, with projected body sizes and bite forces reducing access to some resources across incremental average temperature increases. c: The generality (i.e., here, mean number of plants accessible to ants) of leaf-cutter ants across the three colonies in response to incremental average temperature changes.

Although these changes may sound small in this particular network, they can nevertheless carry substantial consequences for the structure of discrete plant-ant interaction networks. If the ants co-occurred with just a few plants, the loss of even one resource can greatly reduce the fraction of available plant species a colony can forage on (the colony degree), and alter the number of shared plant species across colonies (Figure 4). Such changes have several functional implications, such as predatory/herbivorous release of plant species (Rodriguez, 2006), or a reduction of intra-specific competition, a significant pressure in leaf-cutter ants (Rao, 2000). Whether or not the relatively small variation of body size and bite force with temperature results in meaningful differences in network structure depends on the network and ecological context, and cannot be reliably assessed without more detailed data.

These results support the three hypotheses above, but this is not surprising given the data processing involved. Given that the data on which this analysis is based involve reducing bite forces with temperature increases in line with the temperature-size rule, these hypotheses are likely met because of the design of the analysis. The results can, however, be viewed as testable hypotheses for comparison against real-world field-based data. Given the many other variables that will change under climate change and the many parallel drivers of interaction strengths, observational data will likely differ markedly, but these results present an idealised and isolated effect. This may help to identify the dominance of the temperature-size rule as a driver of these changes.

Table S1: A glossary of terms used throughout the manuscript.

| **Term** | **Definition** | **Main field of study** |
| --- | --- | --- |
| Attachment forces | The force required to separate two joined dislike materials. | Biomechanics |
| Biomechanical performance | The effectiveness of morphological structures in producing work, force or power. | Biomechanics |
| Biomechanical traits | Characteristics linked to biomechanical performance. | Biomechanics |
| Biomechanics | The physical laws that underpin animal movement and structure. | Biomechanics |
| Bite force | The force exerted by animal mouthparts during biting. | Biomechanics |
| Dynamic constraints | Determinants of energy output during continuous or rapid movements. | Biomechanics |
| Elastic strain energy | The energy stored in an elastic material. | Biomechanics |
| First principles | The fundamental concepts or assumptions on which theories, experimental systems or methods are based, usually derived from the basic laws of mathematics and physics. | Biomechanics |
| Force capacity | The maximum amount of force that a muscle can produce. | Biomechanics |
| Locomotor capacity | The potential ability of an organism to move in its environment, comprising speed, endurance and agility. | Biomechanics |
| Locomotor performance | The observed ability of an organism to move in its environment, comprising speed, endurance and efficiency. | Biomechanics |
| Material failure | Damage or destruction of a material as a result of mechanical loading. | Biomechanics |
| Musculoskeletal adaptations | Morphological changes to muscle or skeletal structure to adapt to specific tasks or actions over evolutionary or development time scales. | Biomechanics |
| Penetrability | The effort required to penetrate a material. | Biomechanics |
| Power laws | A mathematical relationship in which one quantity varies as a power of another. |  |
| Quasi-static constraints | Determinants of the magnitude of the maximum forces animals can apply in isometric muscle contractions. | Biomechanics |
| Saccade manoeuvres | The rapid simultaneous movement of body parts in the same direction between two or more phases of fixation. | Biomechanics |
| Biological control | The control of biological systems using biological organisms or products (e.g., control of agricultural pests using their predators). | Ecology |
| Competition | Direct or indirect conflict between organisms for resources | Ecology |
| Conservation biocontrol | The promotion of naturally occurring | Ecology |
| Ecological resilience | The capacity of an ecosystem to withstand or recover from disturbances and pressures. | Ecology |
| Ecosystem function | The biological, geochemical and physical processes that occur within an ecosystem. | Ecology |
| Ecosystem services | The direct and indirect contributions ecosystems provide for human wellbeing and quality of life (e.g., pollination, crop protection). | Ecology |
| Epiphytes | Plants that grow non-parasitically on other plants. | Ecology |
| Evolutionary mismatch | Differences in the traits or ecology of species that reduce the likelihood of interaction as a consequence of evolutionary divergence. | Ecology |
| Foraging ecology | The study of how animals search for, obtain and use food resources. | Ecology |
| Interspecific interactions | Ecological interactions that occur between species. | Ecology |
| Invasive species | Non-native organisms that rapidly colonise new environments, often harming native populations, ecosystems or ecosystem services. | Ecology |
| Nutritional cascades | The diffusion of nutrients through ecological networks. | Ecology |
| Phenology | The annual timing of species emergence and activity. | Ecology |
| Phenological match | The alignment of emergence/activity times of different species. | Ecology |
| Phytophagous | Plant-eating. | Ecology |
| Species invasions | The arrival and spread of invasive species. | Ecology |
| Species turnover | Changes in the species present in a given community over time. | Ecology |
| Temperature-size rule | The principle that ectothermic organisms often grow larger in cooler temperatures and smaller in warmer temperatures. | Ecology |
| Trophic interactions | Interactions that comprise consumption of a resource by a consumer (e.g., predator-prey, herbivore-plant). | Ecology |
| Degree | The number of other nodes a node interacts with. | Network science |
| Ecological network | An interaction network constructed using ecological data. | Network science |
| Generality | The ratio of resources to consumers (i.e., consumer interaction richness). | Network science |
| Group-level | Pertaining to a specific level of a network (e.g., consumers vs. resources). | Network science |
| Indirect interactions | The links between nodes separated by two or more interactions. | Network science |
| Interaction/network rewiring | The redistribution of interactions within a network. | Network science |
| Link | The presence or frequency of interactions between organisms. | Network science |
| Link weights/strengths | The quantitative information underpinning links, usually denoting the frequency of interactions | Network science |
| Motifs | Sets of nodes with similar interactions. | Network science |
| Network ecology | The application of network science to ecological systems. | Network science |
| Network layer | Separate overlapping networks, often spatially, temporally or contextually discrete, linked together as a single multi-layer network. | Network science |
| Network science | The study of interconnected complex systems with graph theory. | Network science |
| Network structure | The topology of a network, usually represented using mathematical properties based on graph theory. | Network science |
| Node | The unit of organisation within networks, usually representing species, individuals or functional groups. | Network science |
| Robustness | The rate of secondary extinctions when some nodes/species are removed. | Network science |
| Secondary extinctions | The loss of nodes in a network after the removal of their interaction partners. | Network science |
| Trait-matching | The prediction of interactions based on the complementarity of functional traits. | Network science |
